# Birth and expansion of NRC immune receptors across the largest group of flowering plants

**DOI:** 10.1093/plcell/koae185

**Published:** 2024-06-25

**Authors:** Renuka Kolli

**Affiliations:** Assistant Features Editor, The Plant Cell, American Society of Plant Biologists; Sainsbury Laboratory, University of Cambridge, Cambridge, UK

Asterids constitute a major clade within flowering plants and encompass over 80,000 plant species, including hundreds that are economically important. Many asterids provide food, spices, and medicines. Phylogenetically, asterids are grouped into 4 clades: Lamiids (e.g. coffee, sweet potato, olive, rosemary, and periwinkle), Campanulids (e.g. carrot, sunflower, and lettuce), Ericales (e.g. tea, kiwifruit, and Brazil nut), and Cornales (e.g. dogwood and hydrangea). The Solanaceae family, belonging to Lamiids, includes major agricultural crops, such as potato, tomato, and pepper. Pathogens and pests attacking these crops substantially reduce yields and qualities. Improving plant disease resistance through breeding is therefore an important strategy for minimizing yield losses.

Plant innate immunity involves intracellular immune receptors called nucleotide-binding domain and leucine-rich repeat-containing receptors (NLRs), which mediate recognition of pathogen effectors and restrict pathogen spread via a form of programmed cell death called the hypersensitive response (HR). While some NLRs function as singletons, many NLRs functionally specialized into either sensor NLRs, for recognizing pathogen effectors, or helper NLRs, for initiating the immune signaling that leads to HR. The functionally linked sensor and helper NLRs can also be genetically linked in pairs or clusters. In Solanaceae species, the NLR-required for cell death (NRC) superclade, comprising the NRC family of helper NLRs (NRC or NRC-H) and their phylogenetically related sensor NLRs (NRC-S), confers immunity against viruses, bacteria, oomycetes, nematodes, and insects (Wu et al. 2017). However, the evolution and function of the NRC network beyond Solanaceae has not been assessed in depth previously. In this issue, 2 groups address this knowledge gap by providing insights into a wider NRC network covering asterids: one focused on conservation, while the other explored diversification within this network.

**Toshiyuki Sakai and coauthors** (Sakai et al. 2024) performed phylogenetic analyses on the NRC superclade genes of various asterid species and found that a highly conserved NRC-H subclade, termed NRC0, contains just 1 or 2 genes from each representative species. In contrast, nonconserved NRC genes were found to have expanded, especially in Lamiids. The NRC-Ss that are phylogenetically and genetically linked to NRC0 were identified as NRC0-dependent sensor candidates (NRC0-S). The authors hypothesized that the NRC network in asterids likely originated from an ancestral NRC-S/NRC-H pair, resembling an NRC0-S/NRC0 pair, before the Caryophyllales and asterid lineages split. (Fig. A). In support of the hypothesis, they validated the functional link of NRC0-S/NRC0 pairs from 4 species belonging to the major asterid subgroups, Campanulids and Lamiids, by HR assays in the *Nicotiana benthamiana* transient expression system. They solidified the functional link of the tomato NRC0-S/NRC0 pair by demonstrating that the NRC0-S activation shifted the paired NRC0 to a higher molecular-weight complex using blue native polyacrylamide gel electrophoresis. Hence, NRC0 appears to form a resistosome-like complex to induce HR, similar to ZAR1, the most widely conserved NLR in flowering plants (Bi et al. 2021; Adachi et al. 2023).

**Figure:**
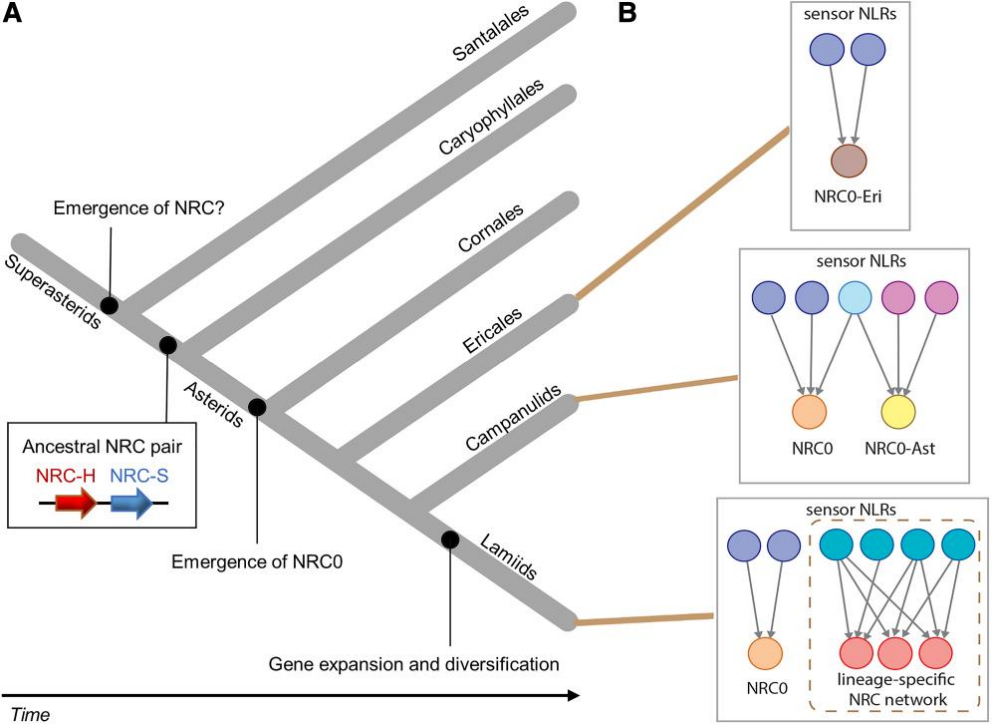
Origin and evolutionary trend of the NRC network in asterids. **A)** An evolutionary model of asterids over 125 million years showing the putative origin of NRC0. **B)** Hierarchy of varied NRC network complexity in the connected lineages of (A). Within each box, the sensor NLRs on top signal through the pointed helper NLRs below to induce HR. Adapted from Sakai et al. (2024), Figure 9 and Goh et al. (2024), Figure 8.

Complementary to the above work, **Foong-Jing Goh and coauthors** (Goh et al. 2024) used similar approaches and determined that the NRC network in Campanulids and Ericales is small, with NRC superclade members making up only 1% to 14% NLRs in these groups, while that in Lamiids dramatically diversified, with NRC superclade members making up 40% to 89% of all NLRs. In addition to the NRC0 subclade identified by Sakai et al., Goh et al. found 2 conserved NRC0 subclades, one specific to Ericales and the other specific to Asterales within Campanulids. Corresponding to the NRC network expansion, the sensor/helper functional linkages in Ericales and Campanulids are simple, whereas those in Lamiids are highly complex (Fig. B). Goh et al. performed many functional analyses to determine the NRC-S/NRC-H compatibility within certain species and across different species of asterids. Their results indicate that the conserved NRC0 members are partially interchangeable, whereas the diversified NRCs in Lamiids show very limited interchangeability.

Immune receptors in plants are ever evolving to compete in the plant-pathogen arms race. Sakai et al. and Goh et al. map out the evolutionary trajectory and functional complexity of the NRC network in asterids from a putative ancestral NRC-H/NRC-S pair that is genetically and functionally linked to an immensely expanded NRC network with many genetically dispersed and complex functional links (Fig.). Moreover, they provide insights into potential transferability of disease resistance mechanisms across asterid species. These findings are a major step toward predicting and overcoming restricted taxonomic functionality of NLRs and open doors to interspecies resistance gene transfer for disease resistance breeding of multiple crop species.
